# Nasopharyngeal malignant melanoma masquerading in the gallbladder: the importance of histological assessment

**DOI:** 10.1308/rcsann.2023.0064

**Published:** 2023-11-20

**Authors:** R Nagra, S Zaman, AYY Mohamedahmed, A Torrance

**Affiliations:** Sandwell and West Birmingham Hospitals NHS Trust, UK

**Keywords:** Gallbladder, Metastatic melanoma, Gallbladder cancer, Metastasis, Cholecystitis, Cholecystectomy

## Abstract

Metastatic melanoma of the gallbladder is a rare entity that is often diagnosed late, leading to a poor prognosis. The disease may present insidiously as acute cholecystitis or remain asymptomatic. Optimal management remains unclear but surgical resection is considered the mainstay of treatment for this condition. We report the case of a 47-year-old man who suffered a protracted course of generalised abdominal symptoms eventually culminating in a diagnosis of acute cholecystitis. Following an emergency laparoscopic cholecystectomy, the histology revealed a melanoma with an unknown primary. Subsequently this was traced to the nasopharynx. Because of the presence of concurrent liver metastasis, systemic immunotherapy with palliative intent was commenced following a multidisciplinary team discussion. This case highlights the importance of sending clinical specimens for histological analysis. We argue against selectively choosing which specimens to send for histology because radiological and/or intraoperative macroscopic inspection of resected tissue alone can result in a missed diagnosis.

## Background

Cutaneous melanoma is an aggressive cancer that is known to metastasise commonly to regional lymph nodes, the lungs, liver, brain and colon. However, spread to the biliary tract and the gallbladder is extremely rare.^1,2^ Paradoxically, despite its rarity, malignant melanoma is the most common primary malignancy metastasising to the gallbladder, accounting for approximately 50% of all cases.^3^

Primary gallbladder malignant melanoma is an even rarer clinical entity^4^ and was first described by Hatanaka *et al*.^5^ They described six melanoma lesions in the gastrointestinal (GI) tract and viscera of a 51-year-old man, including one in the gallbladder.^5^ In a study of 10,500 patients, Dong *et al* found a single case of primary malignant melanoma and a further 19 cases of metastatic melanoma of the gallbladder.^4^

Patients with metastatic melanoma of the gallbladder often present in the fourth or fifth decade of life.^6^ Typically, the condition remains asymptomatic or presents insidiously as acute cholecystitis.^1,7^ The aetiology of the latter probably occurs secondary to a ‘mass-effect’ of the tumour occluding the cystic duct resulting in the onset of infection and inflammation.^4^ Diagnosing this condition is typically reliant on a multimodal approach of radiological imaging and histological assessment following surgical resection.^7^

Primary gallbladder malignant melanoma macroscopically is often described as a relatively large tumour with a polypoidal appearance. By contrast, metastatic melanoma of the gallbladder histologically appears smaller, multifocal, flat and subepithelial.^8^

Given the rarity of metastatic melanoma of the gallbladder the optimal management strategy remains challenging and unclear.^2^ Surgical resection of involved tissue including cholecystectomy is considered the mainstay of melanoma management.^4,8^ In addition, patients may be offered adjuvant treatment following multidisciplinary team (MDT) discussion including chemotherapy, immunotherapy or immunochemotherapy.^8^

However, curative treatment is often not possible in cases of metastatic melanoma of the gallbladder owing to the frequent concurrent involvement of other tissues/organs.^2,4^ Generally, the prognosis is considered poor, with a median survival time of 6–9 months.^4,5^

We report a rare case of metastatic melanoma of the gallbladder presenting as acute cholecystitis. We aim to highlight this highly unusual presentation and stress the importance of routine histological examination of resected tissue.

## Case history

A 47-year-old Caucasian man presented to the emergency department with recurrent episodes of abdominal symptoms. On this occasion, the patient complained of radiating epigastric pain and bouts of vomiting. An electrocardiogram and laboratory tests (troponin) ruled out an acute cardiac event.

The patient’s comorbidities included anxiety, depression, hypertension, gastro-oesophageal reflux disease, bilateral conductive hearing loss, and a previous myocardial infarction requiring primary coronary intervention.

Following admission, an ultrasound scan of the abdomen revealed an over-distended gallbladder containing mixed echo material with anechoic areas. These features were suggestive of tumefactive sludge or a large adenomatous polyp and although considered suspicious, malignancy was thought to be unlikely.

A computerised tomography (CT) scan of the abdomen/pelvis revealed a distended thick-walled gallbladder containing no calcified stones and no pericholecystic collections radiologically suggesting acute cholecystitis. The patient was managed conservatively and discharged with follow-up arranged with the upper GI surgeons.

However, he re-presented several days later with non-resolving right-sided abdominal pain and vomiting with raised inflammatory markers. On examination he was markedly tender in the right upper quadrant and epigastrium with a positive Murphy's/Sweeney's sign.

After a trial of intravenous fluids, broad-spectrum antibiotics and analgesia he was listed for emergency surgery. Laparoscopy findings included a distended, tense, oedematous and thick-walled gallbladder. Technical intraoperative difficulties and the marked inflammation and extensive adhesions eventually resulted in a sub-total cholecystectomy being performed. Further inspection of the gallbladder revealed sludge, necrotic mucosa and approximately 80ml of haemorrhagic fluid. This was thought to be consistent with acute haemorrhagic cholecystitis and the resected specimen was sent for histological analysis. The rest of the laparoscopy was unremarkable with no gross pathology identified.

Histological findings revealed acute haemorrhagic cystitis, no gallstones, multifocal tumour obliterating the neck of the gallbladder and further tumour deposits in the body. The tumour was characterised by focally necrotic sheets of neoplastic epithelioid cells demonstrating extensive pigmentation.

Immunohistochemical staining was negative for pancytokeratin, showing diffuse positivity for SOX10 (a transcription factor and sensitive, specific marker for melanoma) and patchy/weak staining for S100 (low-molecular mass proteins and immunohistochemical markers of malignant melanoma). These findings were in keeping with a poorly differentiated epithelioid neoplasm with features strongly suspicious of malignant melanoma.

**Figure 1:**
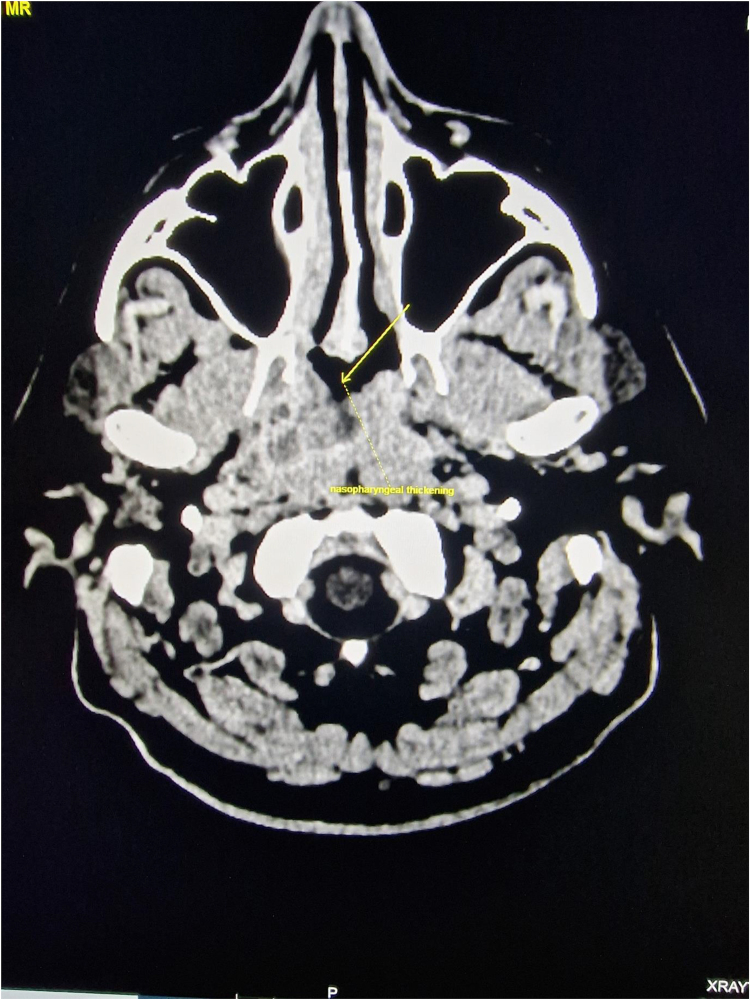
Contrast-enhanced computerised tomography scan image at the level of the nasopharynx showing heterogeneous nasopharyngeal soft tissue thickening

Extensive clinical assessment by the dermatology team revealed no pigmented or suspicious skin lesions. A whole-body staging CT scan revealed a right-sided soft tissue thickening in the nasopharynx (Figure 1) and a 3.5cm×2.3cm liver subcapsular mass close to the resected gallbladder bed. The patient was subsequently referred to the local tertiary centre for specialist melanoma treatment. Examination under anaesthesia of the nasopharynx revealed a large, ulcerated lesion with characteristics of melanoma.

MDT discussion concluded the sino-nasal mucosa of the nasopharynx as being the primary melanoma site with secondary spread to the liver and gallbladder. Given the nature of the disease, the patient is currently under follow-up being treated with palliative systemic immunotherapy (ipilimumab and nivolumab).

## Discussion

Metastatic melanoma of the gallbladder is often diagnosed late, inevitably resulting in a poor prognosis.^4,9^ Traditionally, surgeons have advocated for the approach of sending all gallbladder specimens for histological assessment.^10^ This is done for diagnostic reasons, incidental cancer detection, audit, research and future treatment planning.^10,11^ Siddiqui *et al* found six (2.7%) patients (*n* = 220) with incidental gallbladder adenocarcinoma of varying differentiation following routine histological assessment.^12^ Lohsiriwat *et al* reported 4,317 gallbladder specimens, with benign neoplasms being detected in 16 (0.37%) patients and malignant gallbladder cancers in 27 (0.63%).^11^

Although neither study identified gallbladder malignant melanoma, they nevertheless concluded that routine histological assessment of all gallbladder specimens is justified to prevent the devastating consequences of undiagnosed malignancy.^11,12^

More recently, a move away from this traditional approach in favour of selective histological gallbladder assessment has been proposed.^10,13^ This is due in part to financial constraints on healthcare systems relating to processing costs of resected tissue specimens along with a finite pool of specialist histopathologists.^10^ In the UK, histopathologists have reported an increased workload/clinical demands, rising reporting standards, complex cases and increased sub-specialisation.

Often there is a mismatch between surgeon and histopathology workforces, leading to the overstretching of limited resources.^14^ Only in cases in which gallbladder cancer is suspected on imaging, intraoperative assessment or the patient possesses high-risk features, should histological analysis be considered.^13,14^

However, this approach may not be clinically sound. Siddiqui *et al* found no macroscopic changes evident of malignancy during intraoperative assessment of resected specimens.^12^ Furthermore, no patient presented with clinical features typically associated with underlying malignancy; instead, 92% had features of cholecystitis.^12^ In metastatic melanoma of the gallbladder, this may also be the case.^1,7^

However, Dix *et al* reported five gallbladder cancers in a cohort of 1,308 specimens all demonstrating macroscopic evidence of malignancy. They concluded routine histological assessment was unnecessary because malignancy could be diagnosed through a combination of imaging and intraoperative findings.^14^

Intraoperative findings in the case reported here did not reveal any macroscopic features suspicious of malignancy. Instead, a haemorrhagic, distended gallbladder with necrotic material was found.

Metastatic malignant melanoma of the gallbladder is typically described as being multifocal, flat and subepithelial.^8^ In our case report, suspicion of malignancy was raised on the ultrasound scan but was indeterminate and not diagnostic. Relying solely on radiological imaging and/or intraoperative gallbladder inspection to select suitable specimens for histology may increase the likelihood of missing potential malignancies.

Lastly, prognosis of metastatic gallbladder malignant melanoma is poor owing to late diagnosis and a high likelihood of concurrent metastasis.^15^ Long-term disease-free survival occurs in only 1%–2% of patients. Furthermore, on autopsy, metastatic gallbladder malignant melanoma has been found in up to 15% of patients, often owing to the asymptomatic nature of this disease.^15^

Based on these findings we recommend routine histological assessment of all resected gallbladder specimens to detect incidental cancers, including melanomas, until more-sensitive and cost-effective imaging strategies are able to replace microscopic evaluation.^16^

## Conclusions

Metastatic gallbladder malignant melanoma is a sinister condition with an unfavourable prognosis. Our case highlights the atypical nature of this entity. Intraoperative assessment revealed no gross features typical of this condition and it was difficult to differentiate from other causes of acute cholecystitis. On histological confirmation, owing to its late presentation, the patient has been given a palliative prognosis. Although we accept metastatic gallbladder malignant melanoma is rare there is a need to detect the disease early to improve outcomes. Intraoperative assessment is unreliable, as evidenced by our own findings. Therefore, to prioritise early detection we argue the need for routine histological analysis of all resected gallbladder specimens.
